# Minocycline in Major Depressive Disorder: And overview with considerations on treatment-resistance and comparisons with other psychiatric disorders

**DOI:** 10.1016/j.bbih.2021.100335

**Published:** 2021-08-24

**Authors:** Maria Antonietta Nettis

**Affiliations:** a King’s College London, Institute of Psychiatry, Psychology and Neuroscience, Department of Psychological Medicine, London, UK; b National Institute for Health Research Mental Health Biomedical Research Centre, South London and Maudsley NHS Foundation Trust and King’s College London, London, UK

## Abstract

Evidence on the link between the immune system and Major Depressive Disorder (MDD) has led to explore antidepressant properties of anti-inflammatory drugs. Among these, minocycline has been identified as a potential novel treatment for MDD, in particular for treatment-resistant depression. The aim of the current paper is to review current pre-clinical and clinical evidence on the antidepressant efficacy of minocycline. The review includes considerations on the role of both peripheral and central inflammation in the response to minocycline and comparisons of minocycline efficacy across different psychiatric disorders (i.e., unipolar depression, bipolar depression, and schizophrenia).

## 1 Introduction

Several pharmacological, psychological and neurostimulation treatments are currently available for Major Depressive Disorder (MDD), and when first-line treatments fail, a number of next-step alternatives can be trialled including combination of antidepressants and augmentation with other psychotropic medications and psychotherapy. However, a significant proportion of patients still do not achieve sustained remission, despite serial treatments (McAllister-Williams et al., 2020). Patients who belong to this category (approximately 30% of those suffering from MDD) clearly have “Difficult-To-Treat” depression, more generally referred to as “Treatment-Resistant Depression “(TRD) (Gaynes et al., 2020). Overall, TRD poses a challenging presentation to secondary and primary care clinicians (Rush et al., 2006) and accounts for the highest direct and indirect medical costs among MDD patients (Gibson et al., 2010). To address this important issue, research in the last few years has tried to identify clinical and biological predictors of poor response, which could be theoretically used to personalize treatment practice and escalate sooner the identified patients through more assertive treatment algorithms.

Among biological predictors, the increased activation of the immune system has been found consistently associated with the development of MDD and with TRD. Indeed, depressed patients show increased levels of pro-inflammatory immune markers (Osimo et al., 2020). More interestingly, a recent meta-analysis has found that a composite biomarker of inflammation (including Tumour Necrosis Factor (TNF), Interferon (IFN)α or IFNβ, Interleukin (IL)-1α or IL-1β, IL-6 and C-reactive protein (CRP) was higher in patients with subsequent no-response to antidepressants compared with responders (Strawbridge et al., 2015). Additional evidence has shown higher levels of CRP in 45% of MDD patients not responding to standard antidepressants (Miller and Raison, 2016) and in TRD patients compared with treatment responders (Chamberlain et al., 2019) (Cattaneo et al., 2020). Finally, levels of CRP> 3 mg/L, corresponding to low-grade inflammation, not only are present in approximately 30% of MDD patients (Osimo et al., 2019), but have been specifically associated with Treatment-Resistance (Chamberlain et al., 2019). Taken together, these data suggest that targeting inflammation could be a promising strategy to improve depressive symptoms, particularly in those who do not benefit from standard antidepressant treatment. Therefore, research on the antidepressant effect of anti-inflammatory agents has attracted increasing attention (Kohler et al., 2016).

In this paper, I will focus on one particular anti-inflammatory medication, minocycline, a tetracycline antibiotic which showed antidepressant properties in both animals and humans. After describing minocycline putative mechanisms of action, I will review the pre-clinical and clinical evidence on its efficacy in MDD and the most recent findings on its efficacy in TRD. I will also compare results on the efficacy of minocycline across different psychiatric disorders.

### 1.1 Minocycline mechanisms of action

Minocycline is a second-generation, semi-synthetic tetracycline that has been used for over 30 years because of its antibiotic properties against gram-positive and gram-negative bacteria. It is mainly used for the treatment of acne vulgaris and some sexually transmitted diseases (Garrido-Mesa et al., 2013a). In comparison with other anti-inflammatory agents (e.g., Non-Steroidal Anti-Inflammatory Drugs or cytokine antagonists), minocycline has the advantage of good penetration into the central nervous system (CNS) through the Blood-Brain Barrier, which accounts for its important neuroprotective ability (Soczynska et al., 2012). This is particularly important because the inflammatory cascade leading to depression can involve pathways at the interface between the peripheral immune system and the CNS (Nettis and Pariante, 2020) (Enache et al., 2019). Therefore, agents targeting not only peripheral, but also central inflammation are more likely to show efficacy in immune-related depression.

Minocycline has broader anti-inflammatory properties than other anti-inflammatory agents, due to its inhibitory actions on mechanisms relevant for inflammation-induced depression, like the kynurenine (KYN) and the p-38 pathways.

In the KYN pathway, inflammation leads to the activation of indoleamine 2,3-dioxygenase (IDO), a key enzyme in the metabolism of the serotonin precursor, tryptophan. This results in a reduction of serotonin levels and an increase in neurotoxic metabolites (Roman and Irwin, 2020).

Through the p-38 pathway, inflammatory processes lead to an increased expression and function of the serotonin transporter, resulting in a reduction of serotonin in the synaptic space (Miller and Raison, 2006).

Therefore, of particular relevance is the ability of minocycline to inhibit both the IDO and the p-38 components of inflammation-induced depression (Rojewska et al., 2018).

Moreover, evidence supports minocycline antioxidant properties and its abilities in modulating glutamate and monoamine neurotransmission (better described in paragraph 1.2) (Hashimoto and Ishima, 2010). For example, minocycline appeared to increase dopamine levels in the amygdala and reduce glutamate levels in the hippocampus in animal models (Arakawa et al., 2012) (Soczynska et al., 2012).

Further mechanisms that may be involved in minocycline’s anti-inflammatory, immunomodulatory and neuroprotective effects include 1) inhibitory effects on the activities of key enzymes for the immune response, like inducible Nitric Oxide Syntashe (iNOS), Matrix Metalloproteinases (MMPs) and Secretory Phospholipase A 2 (sPLA_2_); 2) antiapoptotic abilities via inhibition of caspase-1 and caspase-3 activation; enhancement of antiapoptotic Bcl-2 family proteins and inhibition of poly(AD-ribose)-polymerase (PARP)-1 (Garrido-Mesa et al., 2013b).

### 1.2 Minocycline and microglia

Interestingly, minocycline has been suggested to be an inhibitor of microglial activation (Soczynska et al., 2012). Microglia are the brain resident macrophages and provide the main form of adaptive immune response in the CNS: they influence cell proliferation and survival depending on the inflammatory state (Mondelli et al., 2017) In response to harmful stimuli, microglial cells undergo a number of changes (Walker et al., 2014) including production of pro-inflammatory cytokines and the expression of several cell surface antigens. On the other hand, neuroprotective actions of microglia include secretion of neurotrophic factors involved in cellular repair and in the recruitment of immune cells into the CNS (Block et al., 2007). The difference between these 2 microglial actions has led to the distinction between the M1 -pro-inflammatory- and the M2 -anti-inflammatory- phenotype. However, this view has been recently challenged, as another hypothesis is that microglial phenotype might vary along a continuum (Ransohoff, 2016).

Microglial activation associated with a pro-inflammatory state has been detected in animal models of depression (McKim et al., 2016) (Zheng et al., 2015). Activated microglia can impair neuroplasticity by inhibiting the proliferation of neural progenitor cells, and by inhibiting neurite outgrowth in cortical neurons. These effects can be mitigated by minocycline neuroprotective and antiapoptotic properties and also via direct suppression of microglial activation/proliferation, and inhibition of subsequent inflammatory mediator synthesis (Soczynska et al., 2012).

However, the exact molecular mechanisms behind minocycline activity on microglia are not completely clear.

In primary rat microglial cells, minocycline showed the ability to decrease IFNγ-induced MHCII expression. This decreased antigen presentation capacity of CNS resident microglia, a mechanism that may underlie the effects of minocycline in CNS infectious diseases (Nikodemova et al., 2007).

Another possible mechanism might be the inhibition of the NF-KB pathway. In fact, in murine microglia-derived cell lines (BV-2) minocycline prevented the degradation of the inhibitory subunit of IKBα, thus reducing NF-KB translocation to nucleus and its activation (Nikodemova et al., 2006). This resulted in decreased transcription of proinflammatory mediators, such as cytokines, Cyclooxygenase (COX-2) and iNOS. Also, minocycline was shown to inhibit NF-KB binding to DNA in human immunodeficiency virus (HIV)-1-infected microglia (Si et al., 2004).

Minocycline could also block Toll-like-receptor (TLR)-2 surface expression, as suggested by work with BV-2 microglial cells and with microglia isolated from adult mice brains (Garrido-Mesa et al., 2013a). Toll-like receptors (TLRs) are components of the host innate immunity that are abundantly expressed in microglia and mediate microglial transition to an anti-inflammatory state (Facci et al., 2014).

Of note, minocycline effect on microglia is part of its ability to regulate proliferation and activation of several immune cells. This includes direct effects on T cells, monocytes and macrophages, neutrophils and B cells, resulting in the inhibition of these cells proliferation, reduction of pro-inflammatory cascades and reduction of chemotaxis (Garrido-Mesa et al., 2013b).

### 1.3 Pre-clinical evidence of minocycline antidepressant effect

Animal models of depression can be helpful to explore the neurobiological mechanisms underlying depressive symptoms, such as changes in cognition and behaviour. These models are mostly based on stress or on biological causation.

On the one hand, stress-induced models are probably the most commonly used. They have a strong construct validity given that it is well known that stress can trigger depressive-like symptoms development (Pittenger and Duman, 2008).

On the other hand, models based on biological causation (e.g., immune challenges like lipopolysaccharide (LPS) and interferon (IFN)-alpha, bulbectomy, genetic manipulation) rely on physiological alterations. These alterations include factors involved in the HPA axis, the neuroinflammation system, and also neurotransmission.

A recent meta-analysis by Reis and colleagues (Reis et al., 2019) reviewed preclinical evidence on the antidepressant effect of minocycline comparing 22 studies, of which 11 conducted in mice and 11 in rats. Preclinical models of MDD included chronic stress, induced sickness, olfactory bulbectomy-induced depression, and induced type-1 diabetes.

The different studies assessed immobility-based depressive-like behaviour with the forced swim test, the tail-suspension test, and the open field test. Anhedonia-based depressive-like behaviour was assessed with the sucrose preference test. Finally, one study also assessed social avoidance. Overall, minocycline administration, compared with vehicles, significantly reduced depressive-like behaviours in both rats (Standardized Mean Difference (SMD) = −1.22) and mice (SMD = −0.97). Separate analyses revealed that minocycline significantly reduced both immobility-based (SMD = −1.17) and anhedonia-based (SMD = −0.78) outcomes.

Notably, according to this meta-analysis, minocycline showed a significant antidepressant effect among animals that were already experimentally stressed or diseased before they faced the depression assessment protocol (e.g., the forced swimming or prolonged tail suspension). By contrast, minocycline showed no significant impact upon animals that entered the challenging depression paradigm in an otherwise healthy state. This suggests that the experimental models employed by the different studies (i.e. chronic stress, provoked inflammation/sickness, olfactory bulbectomy) were all associated with a significant increase in depressive-like behaviour.

In terms of comparisons between experimental models of depression, those including the administration of an immune challenge, like LPS, seemed to show a greater Effect Size (ES) than their counterparts based on psychosocial stress ((Chijiwa et al., 2015), ES 2.39 vs 0.06, respectively).

Interestingly, two studies by Saravi and colleagues showing large ESs (SMD >2) suggested an anti-depressant efficacy of minocycline in depression models involving the nitric oxide (NO)/cyclic GMP pathway (Saravi et al., 2016). This confirmed that minocycline psychotropic and neuroprotective effects might go beyond its anti-inflammatory signalling.

Furthermore, as mentioned above, minocycline has been suggested to target the monoaminergic system as well. In a learned helplessness (LH) rat model of depression, minocycline exerted its antidepressant-like effects by increasing dopamine and 3,4-dihydroxyphenylacetic acid (DOPAC) levels in the amygdala.

Moreover, minocycline proved to attenuate decreases in serotonin, dopamine and their transporters following psychotomimetic substance administration in mice, rats and monkeys (Soczynska et al., 2012).

#### 1.3.1 Effect on the CNS

Animal studies also specifically highlighted minocycline ability in reducing central inflammation. For example, minocycline proved to block the release of cytokines and the activation of the KYN pathway in the CNS after LPS administration in mice (O’Connor et al., 2009). Moreover, Burke and colleagues showed that chronic administration of minocycline reduces the expression of CD11b, a marker of microglial activation, and of the pro-inflammatory cytokine IL-1β, in the prefrontal cortex of rats subjected to olfactory bulbectomy (Burke et al., 2014). Another study showed that minocycline ameliorates IFN-α-induced microglial activation and depressive-like behaviour (Zheng et al., 2015). Of note, models of chronic stress and those based on repeated immune challenge administration were associated with similar neuroimmune and behavioural presentations, such as increased expression of TNF-alpha mRNa (Guan et al., 2015), microglial activation, reduced hippocampal neurogenesis and increased immobility (Zheng et al., 2015) (Bassett et al., 2021).

#### 1.3.2 Effect on the gut microbiome

Finally, minocycline might exert its neuroprotective and antidepressant action through a modulation of the gut microbiome. Indeed, chronic minocycline treatment (for 4 weeks) inhibited hippocampal neuroinflammation and altered species abundance and metabolites of gut microbiota in mice exposed to unpredictable chronic mild stress (CUMS) (Yang et al., 2020). In the same study, minocycline treatment ameliorated intestinal barrier disruption and reduced the bacteriological indexes, such as diamine oxidase, C-reaction protein, and endotoxin in peripheral blood of CUMS mice. These data suggest that the antidepressant mechanism of minocycline treatment could be due to the combined action of neuroinflammation and gut microbiota modulation.

A study by Schmidtner (Schmidtner et al., 2019) and colleagues found a similar result in rats, with minocycline administration being associated with a reduction in microglial number in the prefrontal cortex, and with changes in microbial composition which could contribute to the observed antidepressant effect. The same study also suggested that minocycline alleviates depressive-like behaviour only in male rats with high anxiety-like behaviour, indicating that the antidepressant effect of minocycline could be sex- and trait-dependent.

Overall, these findings indicate that minocycline might induce a perturbation of the gut microbiome. Whether such an impact could support minocycline antidepressant effect or is instead detrimental in the longer term should be further investigated in both animal and human studies.

Overall, the above-mentioned animal studies suggest that minocycline might have antidepressant properties. However, there is an important consideration to make about preclinical models of depression. The limits of modelling complex human disorders in animals (such as rodents) have been previously highlighted, as well as the related risk of false positive results. More specifically, it has been raised that animal models of depression need not to rely merely on behavioural readouts; future studies should aim to meet specific validity criteria and incorporate neurobiological measures, to help understand depression-related changes in the human brain (Harro, 2019). This would facilitate a correct interpretation of results and their generalizability.

### 1.4 Clinical evidence of the antidepressant effect of minocycline and relevant evidence for TRD

Unlike preclinical evidence, the clinical one is much more limited, due to the paucity of studies conducted so far. An initial open-label clinical trial testing the effects of adjunctive minocycline in 25 MDD patients with psychotic features found a significant improvement in depressive symptoms (Miyaoka et al., 2012). Then, two placebo-controlled, randomized Clinical Trials (RCTs) assessed the augmentation therapy with minocycline 200 mg/day in MDD. Of these, one was a 12-week trial with 71 MDD patients and found that minocycline was superior to placebo in improving Clinical Global Impression scores (ES = −0.62), quality of life and functioning, but not depressive symptoms scores (Dean et al., 2017). The second study included 41 TRD patients, and after 12 weeks on minocycline/placebo, found an effect on depressive symptoms, with a larger decrease in the Hamilton Depression Rating Scale (HAM-D-17) scores after minocycline administration compared with placebo (ES: 1.21) (Husain et al., 2017). A third RCT tested the antidepressant properties of minocycline, although it differed from the other studies for the clinical population, including HIV patients with mild-to-moderate depression (N = 46), and administered minocycline for 6 weeks as monotherapy rather than add-on treatment. The study found that minocycline was superior to placebo in improving depressive symptoms measured with the HAM-D-17 (Emadi-Kouchak et al., 2016).

A meta-analysis of these previous RTCs found an overall pooled effect size of 0.78 (SMD) for a potential antidepressant effect of minocycline vs placebo (Rosenblat and McIntyre, 2018). Such effect size is greater than 0.4, which was considered the threshold for a clinically significant response in previous clinical trials of antidepressant treatment employing HAM-D (Faries et al., 2000).

Despite these results seem encouraging, they are still preliminary. Indeed, conclusions are limited by the studies small sample sizes and by their heterogeneity in terms of patients’ population, severity of depression, duration and mode of treatment.

In addition to the afore-mentioned studies, a more recent RCT that I conducted with my research group was the second one specifically investigating patients with TRD (Nettis et al., 2021). The novelty of this study (MINDEP: MINocycline in DEPression) is that, while testing adjunctive minocycline in TRD for 4 weeks, we considered prospectively the baseline inflammatory state of patients as a key factor moderating response to minocycline. This approach was supported by the evidence about anti-inflammatory properties of minocycline and by a previous RCT on the anti-depressant properties of the cytokines-antagonist Infliximab, in patients with TRD. In this trial, secondary results showed that only patients with higher levels of CRP showed improvement with Infliximab, while those with lower CRP levels deteriorated with treatment (Raison et al., 2013).

In the MINDEP study, we considered minocycline efficacy for 4 weeks in a sample of 39 patients selected for elevated CRP (≥ 1 mg/L) and in subsamples of patients with CRP ≥3 mg/L. We found no difference between minocycline and placebo in improving depressive symptoms at week 4 using the threshold CRP = 1 mg/L. However, in secondary, subgroup analysis, we found an association between baseline levels of CRP≥ 3 mg/L (low-grade inflammation) and response to minocycline, such that an increased response to minocycline (measured with HAM-D-17 changes) was found in patients with CRP≥ 3 mg/L compared with others (Cohen-d ES was in the range 1.5-1.9). Moreover, partial responders to minocycline (showing at least 25% symptoms reduction) had higher levels of baseline CRP, and baseline IL-6. MINDEP also included the measurement of inflammatory cytokines and we found that the effect of minocycline on depressive symptoms by week 4 was mirrored by a greater reduction in IFN-γ levels compared with placebo. However, we found no changes in levels of other markers (Nettis et al., 2021).

Another important finding arising from ours and other research groups is that minocycline proved to be overall safe and well-tolerated in humans across studies. The most known and most common side effects, including nausea, vertigo and mild dizziness, tend to occur early after its administration and disappear shortly following therapy discontinuation (Garrido-Mesa et al., 2013a).

According to the available evidence on RCTs, minocycline appeared to be effective in unipolar depression, with medium-large effect sizes associated with relatively small sample sizes (40–70 participants).

To this regard, a comparison with results on minocycline efficacy in other psychiatric disorders could help clarifying what populations of patients are more likely to benefit from this intervention. Indeed, large trials in both bipolar depression and schizophrenia led to negative results, as described below.

### 1.5 Minocycline in other psychiatric disorders

Minocycline has been investigated as augmentation therapy not only in MDD, but also in bipolar depression and schizophrenia. However, data arising from RCTs in these disorders have been conflicting.

In bipolar depression, open label trials (Murrough et al., 2018; Soczynska et al., 2017) and a 6-week RCT on 99 BD patients (Savitz et al., 2018) suggested that minocycline could reduce depressive symptoms. Moreover, in the study by Savitz and colleagues on bipolar depression, responders to minocycline had significantly greater decreases of IL-6 over 6 weeks of treatment when compared with non-responders. On the other side, results from a more recent, larger (n = 266 randomized patients) 12-week RCT published this year (Husain et al., 2020) found negative results.

Similarly, 2 placebo controlled randomized clinical trials in patients suffering from early-phase schizophrenia (Chaudhry et al., 2012; Levkovitz et al., 2010), with 94 and 54 randomized patients, respectively, found that minocycline was superior to placebo in improving negative symptoms (both studies) and executive functioning (Levkovitz et al., 2010). Encouraging results on minocycline ability to ameliorate positive symptoms were limited to specific subgroups (Chaudhry et al., 2012). However, in a third and larger RCT in patients with early schizophrenia (207 randomized patients), minocycline was ineffective in improving any symptom dimension, even in those with increased baseline markers of peripheral inflammation. Furthermore, minocycline administration was not associated with changes in neuroimaging correlates of schizophrenia and in glutamate neurotransmission (Deakin et al., 2018) (Krynicki et al., 2020).

## 2 Discussion

### 2.1 Minocycline and unipolar depression

Currently published findings on the role of minocycline in depression suggest a potential antidepressant effect for this drug with implications for TRD patients. However, it should be considered that a small number of studies are available at the moment, with marked differences in inclusion and exclusion criteria and small sample sizes. Studies differed for ethnic and social background of the observed population, as well as for study design and duration. Finally, while some studies used minocycline as monotherapy, others employed it as adjunctive therapy. The type of depression (i.e., severity, treatment resistance and presence of comorbidity) of the studies was also variable. For example, comparing the two RCTs on TRD, patients in the study by Husain et al. (2017) were exposed to minocycline for 12 weeks, three time longer than patients in the MINDEP study. Moreover, those in the first study showed more severe depressive symptoms than those enrolled in MINDEP (average baseline HAM-D-17 total score >30 as opposed to values < 20 in MINDEP sample). This could also affect the placebo response in the 2 studies, with increased response associated with lower severity (Fournier et al., 2010).

The efficacy of minocycline in patients with MDD comorbid with HIV (Emadi-Kouchak et al., 2016) supports the hypothesis that this medication could be effective in populations with physical conditions associated with altered activity of the immune system. Future research on the antidepressant effect of minocycline could involve patients with autoimmune disorders and inflammatory conditions (such as diabetes, obesity, rheumatoid arthritis, cancer) in comorbidity with MDD.

Currently, 5 more studies on the efficacy of minocycline in unipolar depression are recruiting or have recently completed recruitment (Clinical.Trials.gov). Of these, 3 focus on TRD patients, while one focus on a population of geriatric patients, who are more likely to present with inflammatory conditions (Ferrucci and Fabbri, 2018). Hopefully, more consistent evidence will be available in future years that will fill the current gaps in knowledge.

### 2.2 The predictive role of peripheral inflammation

Levels of baseline inflammatory biomarkers proved to be a promising predictor of response to anti-inflammatories in general and to minocycline in particular. For example, high baseline CRP before treatment has previously been associated with better response in MDD patients to the cytokine inhibitor Infliximab (Raison et al., 2013). Moreover, high basal levels of IL-6 predicted antidepressant efficacy of anti-inflammatory agents, including celecoxib (Abbasi et al., 2012) and minocycline itself, as showed in a 6-week trial in bipolar depression (Savitz et al., 2018).

Interestingly, CRP = ~3 mg/L (low-grade inflammation) can predict immune related physical illnesses in comorbidity with depression (Osimo et al., 2019). CRP levels ≥3 mg/L have also been associated with reduced connectivity within reward related circuits (measured with fMRI) and with alterations of the glutamate metabolism (Haroon et al., 2016), including increased glutamate release in the basal ganglia. This is particularly relevant considering the role played by the glutamatergic system in the pathogenesis of MDD. Indeed, multiple studies found increased glutamate levels in the plasma, Cerebro-Spinal Fluid (CSF) and tissues of individuals with MDD compared with healthy volunteers (Sanacora et al., 2012; Yuksel and Ongur, 2010). Moreover, antidepressants have also be shown to reduce presynaptic glutamate release (Sanacora et al., 2012). Interestingly, minocycline can modulate the glutamatergic neurotransmission, too. This is supported by animal studies showing that minocycline can reduce glutamate release and can depress glutamate transmission in hippocampal neurons, in a dose-dependent manner (Soczynska et al., 2012).

Data regarding the correlation between depressive symptoms improvement and reduction in inflammatory markers are limited and conflicting. In the study by Savitz and colleagues on bipolar depression, participants who responded to minocycline had significantly greater decreases of IL-6 over 6 weeks of treatment when compared with non-responders. Similarly, a correlation between reduced IL-6 and reduced HAM-D scores was found after 6 weeks of celecoxib in patients with MDD, in the study by Abbasi et al. (2012).

By contrast, the MINDEP study did not find a reduction in inflammatory biomarkers following minocycline administration, with the exception of IFN-γ.

Indeed, while the predictive role of baseline CRP and IL-6 is in line with minocycline anti-inflammatory action described in the introduction, the lack of a clear reduction in inflammatory markers in MINDEP is in contrast with evidence from previous studies. However, this might be due to the shorter trial duration in MINDEP (4 weeks).

In summary, due to the paucity and inconsistency of evidence available, it is difficult to draw conclusions on the association between minocycline effect on peripheral inflammation and antidepressant effect. Nevertheless, integrating the measurement of baseline peripheral inflammatory markers in patients’ assessments could still be informative of possible central inflammatory processes, which might be the target of minocycline. Indeed, the levels of peripheral CRP in MDD patients have been associated with the levels of the same marker in the CSF, and plasma CRP>3 mg/L has been associated with higher levels of CSF TNF-alpha and IL-6 soluble receptors, in turn associated with depressive symptoms severity in a recent study by Felger and colleagues (Felger et al., 2020). Therefore, the measurement of baseline inflammatory markers, and in particular CRP, appears to be informative about central inflammatory processes and seems well-suited for guiding immunotherapies in patients with depression.

### 2.3 Comparison with other psychiatric disorders

It is also worth noting that minocycline efficacy might be diagnosisspecific, considering that results about bipolar depression and also negative symptoms of schizophrenia are more conflicting. In particular, a recent, large clinical trial conducted in patients with schizophrenia (Krynicki et al., 2020) found no evidence of an effect of minocycline on symptoms domains, particularly on depressive and negative symptoms. A possible interpretation is that the mechanisms underlying negative symptoms and comorbid depression in psychosis might be very different from those present in MDD and targeted by minocycline. Even the evidence on microglial activation in patients with schizophrenia seems more inconsistent and less robust compared with unipolar depression. This is supported by conflicting results across PET-TSPO studies investigating microglial changes in schizophrenia (Mondelli et al., 2017) (Notter and Meyer, 2017) (Marques et al., 2019).

The interpretation of such inconsistency of findings across psychiatric disorders is either that minocycline efficacy is limited to unipolar depression or that positive findings in unipolar depression are simply a reflection of underpowered analysis. So far, studies in unipolar depression included relatively small samples of patients (<100) and it cannot be excluded that future, larger trials will find negative results.

### 2.4 Tolerability

Minocycline proved to be relatively well tolerated in RCTs conducted so far across different psychiatric disorders. Nevertheless, concerns could be raised that a prolonged anti-biotic administration in patients with psychiatric disorders could lead to the emergence of antibiotic-resistant bacteria. Minocycline has an overall low propensity to produce antibiotic-resistance (Soczynska et al., 2012), but evidence of resistance to this antibiotic, together with resistance mechanisms, have been described (Asadi et al., 2020). Overall, little is known about the development of antibiotic-resistance in patients with psychiatric conditions, where no specific bacteria are targeted by minocycline. Future clinical trials should further investigate this aspect.

## 3 Conclusion

**Fig. 1:**
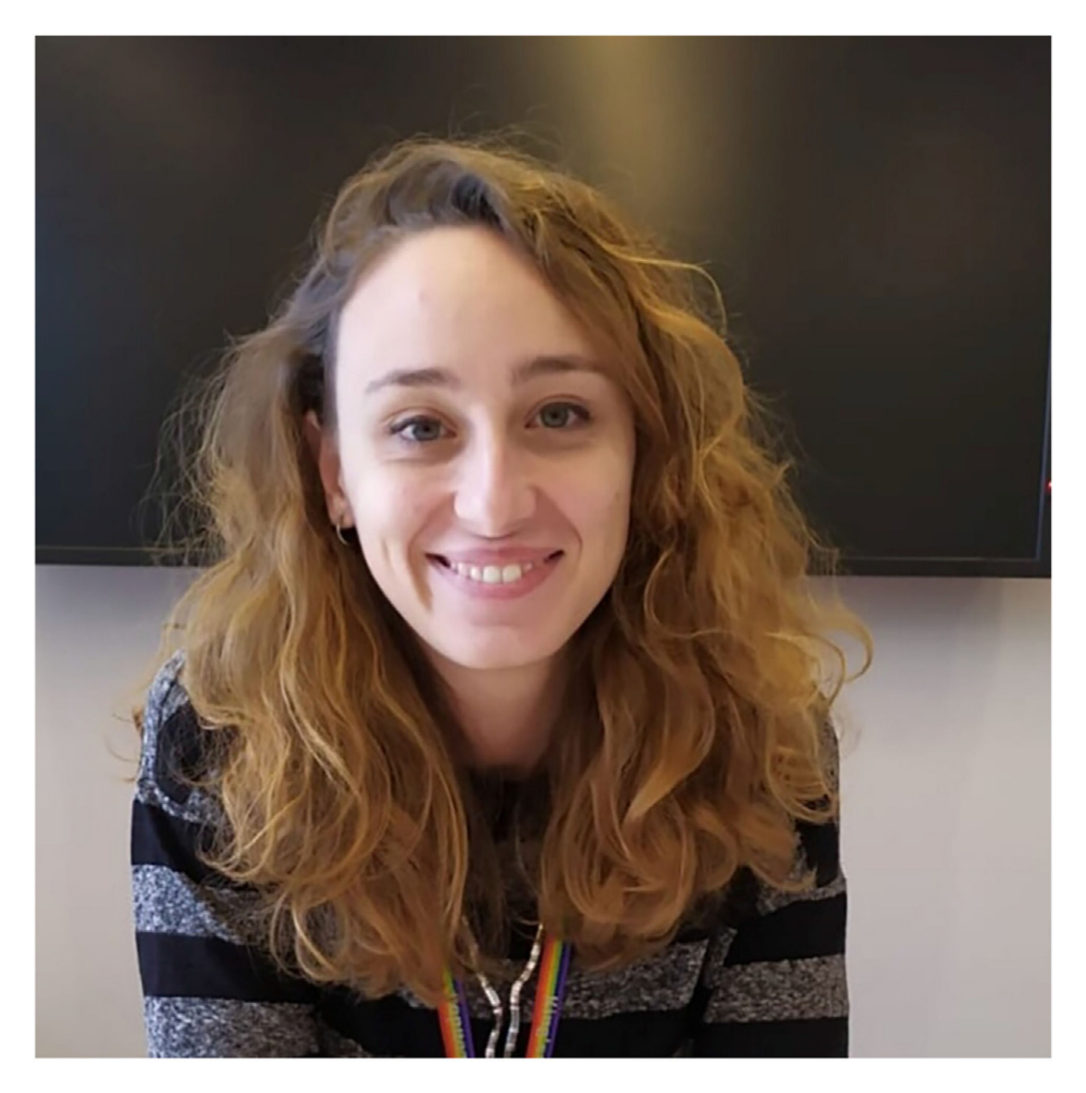
Maria Antonietta Nettis Dr Maria Antonietta Nettis is an academic psychiatrist with a strong interest in the interplay between body and mind in psychiatric conditions. As a psychiatrist, she currently works at the National Psychosis Unit, Bethlem Royal Hospital. As a researcher, she is part of the Research & Development Department committee at South London and Maudsley, NHS Trust, where she contributes to clinical research studies delivery. Dr Nettis has recently completed her PhD in Psychological Medicine at the Institute of Psychiatry, Psychology and Neuroscience, King’s College London. Amongst her research achievements, she was able to demonstrate how a worse immune-metabolic status can predict a poor clinical outcome in first episode psychosis. She also designed a human model of the mechanism of communication between the peripheral and the central immune system, by using PET and MRI to image the neuroinflammatory response to interferon-alpha. Finally, she published data on a RCT with add-on minocycline in patients with treatmentresistant depression and increased levels of inflammation. Currently, Dr Nettis is the local Clinical Principal Investigator for King’s College London in a multi-centre RCT investigating the efficacy of a novel antiinflammatory drug in treatment-resistant depression (**A**ntidepressant **T**reatment with **P**2X7Antagonist). For her research work, she received several awards including the First place Mental Health Foundation Research Prize, from the Royal Society of Medicine (2019) and the Robert Kerwin Bursary from the British Association of Psychopharmacology (2020). Dr Nettis is actively involved in the Institute of Psychiatry network promoting diversity and inclusion and gender equality in STEM.

Current studies provide preliminary evidence for an antidepressant effect of minocycline, particularly in unipolar depression. Minocycline also proved to be generally safe and well-tolerated. However, future larger RCTs are needed to confirm these results. Possible directions could include studies on depressed patients with comorbid physical disorders associated with immune dysregulations (Fig. 1).
